# Supplementary material for: The Additive Values of the Classification of Higher Serum Uric Acid Levels as a Diagnostic Criteria for Metabolic-Associated Fatty Liver Disease
Source: Nutrients. 2022 Aug 31;14(17):3587. doi: 10.3390/nu14173587 (PMC9460100; doi:10.3390/nu14173587)
Supplement: Supplementary file 1 [file nutrients-14-03587-s001.zip › nutrients-1865362-supplementary.pdf]

Table S1. Baseline characteristics stratified by the quartiles of uric acid level in male and female MAFLD patients diagnosed by ultrasonography

Characteristics	Male MAFLD(n=2,345)				*P*	Female MAFLD(n=1,192)				*P*
	Q1 (n=586)	Q2 (n=596)	Q3 (n=577)	Q4 (n=586)		Q1 (n=298)	Q2 (n=304)	Q3 (n=294)	Q4 (n=296)	
Age, years	45.4±12.8	42.8±12.7	41.7±12.3	40.2±13.3	< 0.01	52.8±13.4	51.7±13.1	48.1±13.1	46.2±13.0	< 0.01
BMI, kg/m^2	26.3±2.9	26.7±3.1	27.1±3.5	27.5±3.6	< 0.01	27.5±3.5	27.7±3.6	25.9±3.7	26.9±3.7	< 0.01
SBP, mmHg	129±15	131±15	131±15	132±16	0.02	133±20	132±19	131±16	133±17	0.49
DBP, mmHg	83±10	83±11	84±11	85±12	< 0.01	82±11	82±11	82±10	83±11	0.29
Hypertension, n(%)	190(32.4)	201(33.7)	197(34.1)	226(38.6)	< 0.01	74(24.8)	77(25.3)	84(28.6)	96(32.4)	< 0.01
T2DM, n(%)	142(24.2)	159(26.7)	173(30.0)	188(32.1)	< 0.01	76(25.5)	79(26.0)	83(28.2)	94(31.8)	< 0.01
ALT, U/L	34±26	36±30	44±38	46±36	< 0.01	25±15	29±24	29±19	36±33	< 0.01
AST, U/L	26±16	27±20	30±19	31±26	< 0.01	23±12	28±29	26±19	29±22	< 0.01
GGT, U/L	52±61	62±84	69±103	70±89	< 0.01	39±49	47±83	47±49	61±115	0.01
ALP, U/L	74±23	77±25	80±34	79±32	< 0.01	71±22	78±47	80±28	86±81	0.01
Total cholesterol, mmol/L	5.1±1.1	5.1±1.0	5.3±1.1	5.3±1.2	< 0.01	5.3±1.3	5.4±1.2	5.3±1.2	5.4±1.1	0.86
Triglyceride, mmol/L	2.0±1.4	2.0±1.2	2.0±1.3	2.4±2.1	< 0.01	1.7±1.3	1.8±1.2	1.8±0.9	2.0±1.1	<0.01

HDL-C, mmol/L	1.2±0.3	1.1±0.3	1.1±0.3	1.0±0.2	0.03	1.3±0.4	1.3±0.4	1.3±0.4	1.2±0.3	<0.01
LDL-C, mmol/L	3.2±0.9	3.3±0.8	3.4±0.9	3.4±0.8	0.02	3.3±1.1	3.4±1.0	3.4±0.9	3.4±0.9	0.70
FBG, mmol/L	4.8±2.1	5.0±1.6	5.2±1.3	5.4±1.5	< 0.01	5.1±2.4	5.1±2.2	5.2±1.4	5.4±2.0	0.04
HbA1c, %	6.0±2.0	6.1±1.0	6.3±1.0	6.9±1.3	< 0.01	6.1±2.0	6.2±1.3	6.4±1.6	6.6±1.4	<0.01
Uric acid, μmol/L	316±45	393±11	442±17	552±84	< 0.01	250±40	331±17	383±12	473±90	< 0.01
FIB-4	0.9±0.6	0.9±0.6	0.9±0.5	0.8±0.6	0.31	1.0±0.6	1.0±0.7	0.9±0.7	1.0±0.6	0.20
FIB-4 > 1.0, n(%)	198(33.8)	176(29.5)	159(27.6)	174(30.0)	0.29	84(28.2)	96(31.6)	92(31.3)	96(32.4)	0.79
FIB-4 > 1.30, n(%)	116(19.8)	85(14.3)	104(18.0)	97(16,6)	0.54	44(14.8)	57(18.8)	53(18.0)	56(18.9)	0.64
APRI	0.21±0.14	0.19±0.11	0.26±0.17	0.25±0.09	0.43	0.23±0.09	0.20±0.14	0.26±0.15	0.29±0.17	0.51
APRI⩾0.5, n(%)	26(4.4)	29(4.9)	26(4.5)	28(4.8)	0.67	17(5.7)	14(4.6)	16(5.4)	19(6.4)	0.56
Moderate to severe steatosis, n(%)	180(30.7)	175(29.4)	244(42.3)	300(51.2)	< 0.01	55(18.5)	63(20.7)	51(17.3)	154(52.0)	< 0.01
Severe steatosis, n(%)	28(4.8)	33(5.5)	60(10.4)	91(15.5)	< 0.01	18(6.0)	19(6.3)	14(4.8)	41(13.9)	< 0.01

Category of SUA quartiles

For male: Q1, ≤371 μmol/L; Q2, 372–412 μmol/L; Q3, 413–473 μmol/L; Q4, ≥474 μmol/L.

For female: Q1, ≤300 μmol/L; Q2, 301–359 μmol/L; Q3, 360–406 μmol/L; Q4, ≥407 μmol.

Abbreviation: NAFLD, nonalcoholic fatty liver disease; BMI, body mass index; SBP, systolic blood pressure; DBP, diastolic blood pressure; T2DM, type 2 diabetes mellitus; ALT, alanine aminotransferase; AST, aspartate aminotransferase; GGT, γ-Glutamyl transpeptidase; ALP, Alkaline phosphatase; HDL-C, high-density lipoprotein cholesterol; LDL-C, low-density lipoprotein cholesterol; FBG, fasting blood glucose; HbA1c, glycated hemoglobin; FIB-4, fibrosis-4 index. APRI: the AST to Platelet Ratio Index.

Table S2. Baseline characteristics stratified by the quartiles of uric acid level in male and female MAFLD patients diagnosed by MRI-PDFF

Characteristics	Male MAFLD (n=767)				*P*	Female MAFLD(n=250)				*P*
	Q1	Q3	Q3	Q4		Q1	Q 2	Q3	Q4	
	n=184	n=193	n=189	n=201		n=58	n=62	n=61	n=69	
Age, years	46.8±10.2	45.4±11.4	43.9±11.3	42.0±11.9	< 0.01	53.5±12.2	52.8±12.3	49.6±11.7	47.4±14.8	< 0.01
BMI, kg/m^2	26.0±2.5	27.3±3.4	27.5±3.2	28.1±3.6	< 0.01	25.8±3.0	25.8±3.7	27.3±3.4	28.3±3.7	< 0.01
Waist circumference, cm	89.1±6.9	92.3±8.1	92.6±8.1	94.0±9.0	< 0.01	84.0±6.3	85.5±9.2	89.1±7.4	91.6±8.2	< 0.01
Abodominal obesity, n(%)	85(46.2)	101(52.3)	119(63.0)	128(63.7)	< 0.01	46(79.3)	46(74.2)	54(88.5)	57(82.7)	< 0.01
WHR	0.9±0.05	0.9±0.04	0.9±0.05	1.0±0.05	< 0.01	0.9±0.04	0.9±0.05	0.9±0.05	1.0±0.04	< 0.01
SBP, mmHg	128±15	130±16	132±13	136±16	< 0.01	129±16	130±21	132±14	136±17	0.09
DBP, mmHg	85±11	87±12	87±11	89±12	0.04	84±12	85±11	86±11	88±11	0.10
Hypertension, n(%)	59(32.1)	67(34.7)	67(35.4)	75(37.3)	< 0.01	18(31.0)	20(32.3)	20(32.8)	26(37.7)	< 0.01
T2DM, n(%)	48(26.1)	54(28.0)	56(29.6)	61(30.3)	< 0.01	15(25.9)	18(29.0)	20(32.8)	24(34.8)	< 0.01
ALT, U/L	32±28	36±31	41±31	46±27	< 0.01	24±16	27±17	32±21	35±30	< 0.01
AST, U/L	23±22	28±23	31±22	38±32	< 0.01	22±18	32±25	34±23	35±26.	0.04
GGT, U/L	52±51	67±79	68±82	70±54	< 0.01	47±44	55±61	68±55	80±158	< 0.01

ALP, U/L	72±19	80±17	81±28	81±21	0.03	69±19	78±23	82±35	106±139	0.03
Total cholesterol, mmol/L	5.0±1.0	5.2±1.0	5.2±1.1	5.3±1.1	0.11	5.0±0.9	5.3±1.2	5.0±1.1	5.2±0.9	0.29
Triglyceride, mmol/L	1.7±0.9	1.7±0.9	1.8±1.0	2.3±2.0	< 0.01	1.5±1.0	1.6±1.4	1.9±1.1	2.0±0.8	< 0.01
HDL-C, mmol/L	1.1±0.3	1.1±0.3	1.1±0.2	1.1±0.2	< 0.01	1.3±0.2	1.3±0.2	1.2±0.5	1.0±0.2	0.54
LDL-C, mmol/L	3.0±0.9	3.1±0.8	3.3±0.8	3.4±0.8	< 0.01	3.1±0.7	3.3±0.9	3.0±0.8	3.3±0.7	0.11
FFA, μmol/L	522±186	536±174	550±171	575±182	0.03	536±174	660±216	594±147	658±410	0.03
FBG, mmol/L	5.1±1.0	5.1±1.0	5.1±0.9	5.0±0.9	0.40	5.3±1.4	5.4±1.4	5.3±1.1	5.5±1.7	0.86
HbA1c, %	5.9±1.5	6.0±1.7	6.2±1.9	6.3±2.1	0.02	6.0±1.9	6.1±1.7	6.1±2.0	6.2±1.4	0.36
FINS, μU/ml	10.2±6.5	11.9±7.6	12.7±6.7	13.4±8.7	< 0.01	11.0±5.7	11.9±6.1	13.5±5.9	15.7±6.4	0.28
HOMA-IR	2.5±1.9	2.7±2.7	2.9±1.6	3.0±2.1	0.02	2.7±2.0	2.9±1.9	3.7±5.5	3.3±1.8	0.33
HOMA-IR≥2.5, n(%)	62(33.7)	82(42.5)	88(46.6)	87(43.3)	0.02	23(39.7)	30(48.3)	25(41.0)	37(53.7)	0.09
Uric acid, μmol/L	301±44	387±17	441±16	549±68	< 0.01	256±35	327±16	383±16	458±50	< 0.01
SWE, kpa	6.0±2.7	5.9±3.0	6.3±3.1	6.0±2.2	0.39	6.0±3.4	5.8±3.3	6.2±3.2	6.1±2.5	0.44
SWE≥6.1 kpa, n(%)	85(46.2)	93(48.2)	96(50.8)	99(49.3)	0.28	28(48.3)	31(50.0)	30(61.0)	36(52.2)	0.56
SWE≥7.1 kpa, n(%)	46(25.0)	48(24.9)	49(25.9)	46(22.9)	0.44	14(24.1)	14(22.6)	16(26.2)	15(21.7)	0.36
Liver fat content, %	11.3±5.9	12.9±6.8	14.7±7.6	17.1±8.5	< 0.01	13.3±7.6	12.8±7.8	13.4±7.4	17.8±9.0	< 0.01

Moderate to severe steatosis, n(%)	28(15.2)	40(20.7)	71(37.6)	99(49.3)	< 0.01	17(29.3)	14(22.6)	18(29.5)	32(46.4)	0.03
Severe steatosis, n(%)	15(8.2)	21(10.9)	33(17.5)	60(29.9)	< 0.01	11(19.0)	7(11.5)	7(11.5)	20(29.0)	0.03

Category of SUA quartiles

For male: Q1, ≤371 µmol/L; Q2, 372–412 µmol/L; Q3, 413–473 µmol/L; Q4, ≥474 µmol/L.

For female: Q1, ≤300 µmol/L; Q2, 301–359 µmol/L; Q3, 360–406 µmol/L; Q4, ≥407 µmol.

Abbreviation: MAFLD, metabolic-associated fatty liver disease; MRI-PDFF, magnetic resonance imaging proton density fat fraction; BMI, body mass index; WHR, waist-to-hip ratio; SBP, systolic blood pressure; DBP, diastolic blood pressure; T2DM, type 2 diabetes mellitus; ALT, alanine aminotransferase; AST, aspartate aminotransferase; GGT, γ-Glutamyl transpeptidase; ALP, alkaline phosphatase; HDL-C, high-density lipoprotein cholesterol; LDL-C, low-density lipoprotein cholesterol; FFA, free fatty acids; FBG, fasting blood glucose; HbA1c, glycated hemoglobin;HOMA-IR, homeostasis model assessment of insulin resistance; SWE, shear wave elastography. APRI: the AST to Platelet Ratio Index.

Table S3. Quartiles of uric acid levels and risks of steatosis severity in MAFLD patients estimated with ultrasonography

SUA levels	Unadjusted		Model 1[a]		Model 2[b]		Model 3[c]	
	OR (95% CI)	*P*	OR (95% CI)	*P*	OR (95% CI)	*P*	OR (95% CI)	*P*
**Moderate to severe steatosis**								
**Male**								
Q1 (n=586)	1.00 (Ref)	-	1.00 (Ref)	-	1.00 (Ref)	-	1.00 (Ref)	-
Q2 (n=596)	0.94 (0.73-1.20)	0.61	0.84 (0.65-1.08)	0.17	0.76 (0.58-1.01)	0.06	0.79 (0.61-1.07)	0.08
Q3 (n=577)	1.65 (1.30-2.10)	<0.01	1.50 (1.17-1.91)	<0.01	1.29 (0.99-1.67)	0.06	1.12 (0.86-1.47)	0.41
Q4 (n=586)	2.37(1.86-3.00)	<0.01	2.08 (1.63-2.65)	<0.01	1.74 (1.34-2.25)	<0.01	1.53 (1.17-2.00)	<0.01
**Female**								
Q1 (n=298)	1.00 (Ref)	-	1.00 (Ref)	-	1.00 (Ref)	-	1.00 (Ref)	-
Q2 (n=304)	1.16 (0.77-1.73)	0.48	1.17 (0.78-1.75)	0.45	1.15 (0.74-1.77)	0.54	1.16 (0.75-1.80)	0.51
Q3 (n=294)	0.93 (0.61-1.41)	0.73	0.87 (0.56-1.33)	0.51	1.28 (0.81-2.04)	0.29	1.29 (0.81-2.06)	0.28
Q4 (n=296)	4.83 (3.33-7.00)	<0.01	4.71 (3.24-6.83)	<0.01	7.09 (4.68-10.73)	<0.01	7.26 (4.74-11.10)	<0.01
**Severe steatosis**								
**Male**								

Q1 (n=586)	1.00 (Ref)	-	1.00 (Ref)	-	1.00 (Ref)	-	1.00 (Ref)	-
Q2 (n=596)	1.17 (0.70-1.96)	0.56	0.88 (0.59-1.67)	0.64	0.90 (0.53-1.55)	0.71	0.90 (0.52-1.55)	0.70
Q3 (n=577)	2.31 (1.45-3.68)	<0.01	2.00 (1.25-3.19)	<0.01	1.64 (1.01-2.68)	0.04	1.52 (0.92-2.49)	0.10
Q4 (n=586)	3.66 (2.36-5.69)	<0.01	2.99 (1.91-4.68)	<0.01	2.34 (1.46-3.73)	<0.01	2.08 (1.28-3.36)	<0.01
**Female**								
Q1 (n=298)	1.00 (Ref)	-	1.00 (Ref)	-	1.00 (Ref)	-	1.00 (Ref)	-
Q2 (n=304)	1.04 (0.53-2.02)	0.92	1.06 (0.54-2.07)	0.52	1.02(0.51-2.05)	0.96	1.01 (0.50-2.02)	0.99
Q3 (n=294)	0.78 (0.38-1.59)	0.40	0.68 (0.33-1.42)	0.31	0.99 (0.46-2.11)	0.97	0.99 (0.46-2.14)	0.99
Q4 (n=296)	2.51 (1.41-4.48)	<0.01	2.39 (1.33-4.28)	<0.01	3.01 (1.63-5.58)	<0.01	2.91 (1.55-5.47)	<0.01

Table S4. Quartiles of uric acid levels and risks of steatosis severity in MAFLD patients estimated with MRI-PDFF(LFC≥16.3%)

SUA levels	Unadjusted		Model 1[a]		Model 2[b]		Model 3[c]	
	**OR (95% CI)**	***P***	**OR (95% CI)**	***P***	**OR (95% CI)**	***P***	**OR (95% CI)**	***P***
**LFC≥16.3%**								
**Male**								
Q1 (n=184)	1.00 (Ref)	-	1.00 (Ref)	-	1.00 (Ref)	-	1.00 (Ref)	-

Q2 (n=193)	1.46 (0.86-2.48)	0.17	1.38 (0.81-2.36)	0.24	1.19 (0.69-2.06)	0.53	0.99 (0.56-1.77)	0.99
Q3 (n=189)	3.35 (2.04-5.52)	<0.01	3.07 (1.86-5.08)	<0.01	2.67 (1.60-4.44)	<0.01	2.20 (1.29-3.77)	<0.01
Q4 (n=201)	5.41 (3.32-8.81)	<0.01	4.72 (2.87-7.75)	<0.01	3.96 (2.39-6.56)	<0.01	3.28 (1.93-5.58)	<0.01
**Female**								
Q1 (n=58)	1.00 (Ref)	-	1.00 (Ref)	-	1.00 (Ref)	-	1.00 (Ref)	-
Q2 (n=62)	0.70(0.31-1.60)	0.40	0.70(0.31-1.59)	0.39	0.67(0.29-1.55)	0.59	0.57 (0.23-1.39)	0.57
Q3 (n=61)	1.01(0.46-2.22)	0.98	1.01 (0.45-2.22)	0.99	0.89 (0.40-2.00)	0.78	0.92 (0.38-1.80)	0.84
Q4 (n=69)	2.09(0.99-4.36)	0.06	2.04 (0.97-4.29)	0.06	1.67 (0.77-3.64)	0.20	1.41 (0.61-3.25)	0.42
**LFC≥21.7%**								
**Male**								
Q1 (n=184)	1.00 (Ref)	-	1.00 (Ref)	-	1.00 (Ref)	-	1.00 (Ref)	-
Q2 (n=193)	1.38(0.69-2.76)	0.37	1.24 (0.61-2.51)	0.55	1.05 (0.51-2.14)	0.90	0.89 (0.43-1.87)	0.76
Q3 (n=189)	2.38(1.25-4.56)	<0.01	2.01 (1.04-3.87)	0.04	1.72 (0.88-3.35)	0.11	1.41 (0.71-2.82)	0.33
Q4 (n=201)	4.79(2.61-8.81)	<0.01	3.71 (1.99-6.91)	<0.01	3.06 (1.62-5.78)	<0.01	2.54 (1.31-4.91)	<0.01
**Female**								
Q1 (n=58)	1.00 (Ref)	-	1.00 (Ref)	-	1.00 (Ref)	-	1.00 (Ref)	-

Q2 (n=62)	0.54(0.20-1.52)	0.24	0.53 (0.19-1.50)	0.23	0.48(0.17-1.38)	0.18	0.41 (0.13-1.28)	0.13
Q3 (n=61)	0.51(0.20-1.54)	0.26	0.54 (0.19-1.53)	0.25	0.46 (0.16-1.34)	0.15	0.45 (0.14-1.40)	0.17
Q4 (n=69)	1.74(0.76-4.03)	0.19	1.66 (0.71-3.90)	0.24	1.30 (0.53-3.21)	0.56	1.11 (0.41-2.95)	0.84

[a] Model 1: adjusted for age

[b] Model 2: further adjusted for body mass index , systolic blood pressure.

[c] Model 3: further adjusted for alanine aminotransferase, total cholesterol, fasting blood glucose,

For male: Q1, ≤371 µmol/L; Q2, 372–412 µmol/L; Q3, 413–473 µmol/L; Q4, ≥474 µmol/L.

For female: Q1, ≤300 µmol/L; Q2, 301–359 µmol/L; Q3, 360–406 µmol/L; Q4, ≥407 µmol.

Table S5. Anthropometrical and metabolic characteristic of lean/normal NAFLD and MAFLD patients estimated with MRI-PDFF

Characteristics	Lean /normal NAFLD	MAFLD			$P_{\text{LN vs LM}}$
		Overweight /obesity	Lean/normal weight	Type-2 diabetes	
	n=130	n=646	n=75	n=296	
Age, years	43.0±12.6	40.2±11.9	45.4±12.7	49.7±12.7	0.16
Male, n(%)	79(60.8)	509(78.8)	42(56.0)	216(56.2)	0.50
BMI, kg/m^2	21.6±1.2	27.5±3.2	21.8±1.1	26.7±2.9	0.70
Waist circumference, cm	78.3±5.2	91.6±8.1	79.3±4.9	91.7±8.1	0.34
Abodominal obesity, n(%)	13(10.0)	416(64.4)	13(17.3)	207(70.0)	0.13
WHR	0.9±0.05	0.9±0.05	0.9±0.05	0.9±0.05	0.18
SBP, mmHg	126±17	131±15	130±18	133±17	0.06
DBP, mmHg	82±12	84±12	84±11	83±12	0.04
Hypertension,n(%)	40(30.8)	207(32.0)	30(40.0)	115(38.9)	0.18
ALT, U/L	34±25	39±37	36±29	37±31	0.83
AST, U/L	37±31	28±31	38±36	31±26	0.71

GGT, U/L	57±76	61±62	65±81	74±132	0.47
ALP, U/L	77±21	81±42	79±22	82±38	0.78
Total cholesterol, mmol/L	5.0±1.2	5.2±1.0	5.1±1.2	5.1±1.3	0.71
Triglyceride, mmol/L	1.6±0.9	1.8±1.1	1.9±1.0	2.2±1.2	0.12
HDL-C, mmol/L	1.2±0.4	1.1±0.3	1.2±0.4	1.2±0.3	0.14
LDL-C, mmol/L	3.1±0.8	3.3±0.8	3.1±0.9	3.1±0.8	0.81
FFA, μmol/L	565±184	553±206	576±152	583±192	0.70
FBG, mmol/L	4.8±0.6	4.9±0.6	4.9±0.6	7.9±2.0	0.19
HbA1c, %	5.9±1.8	6.2±2.1	5.9±1.7	7.3±2.9	0.74
FINS, μU/ml	8.8±4.4	12.2±9.9	10.1±4.4	14.7±8.6	0.29
HOMA-IR	1.9±1.0	2.8±2.3	2.2±1.0	5.4±3.5	0.29
HOMA-IR ≥2.5, n(%)	31(23.8)	223(34.5)	28(37.3)	202(68.2)	0.24
Uric acid, μmol/L	375±87	426±99	370±81	401±93	0.70
SWE, kpa	6.0±3.5	6.0±3.0	5.9±3.3	6.2±3.0	0.82
SWE≥6.1 kpa, n(%)	55(42.3)	311(48.1)	30(40.0)	157(53.0)	0.77
Liver fat content, %	11.8±6.9	14.1±7.7	12.4±7.2	15.8±7.9	0.55

Moderate to severe steatosis, n(%)	28(21.5)	187(28.9)	17(22.7)	115(38.9)	0.47
Severe steatosis, n(%)	13(10.0)	93(14.4)	7(9.3)	74(25.0)	0.94

Abbreviation:NAFLD, nonalcoholic fatty liver disease; MAFLD, metabolic-associated fatty liver disease; LN, lean or normal NAFLD; LM, lean or normal MAFLD; BMI, body mass index; WHR, waist-to-hip ratio; SBP, systolic blood pressure; DBP, diastolic blood pressure; T2DM, type 2 diabetes mellitus; ALT, alanine aminotransferase; AST, aspartate aminotransferase; GGT, γ-Glutamyl transpeptidase; ALP, alkaline phosphatase; HDL-C, high-density lipoprotein cholesterol; LDL-C, low-density lipoprotein cholesterol; FFA, free fatty acids; FBG, fasting blood glucose; HbA1c, glycated hemoglobin;HOMA-IR, homeostasis model assessment of insulin resistance; APRI: the AST to Platelet Ratio Index; SWE, shear wave elastography.

Table S6. Characteristic of subtypes of MAFLD patients with SHUA estimated with MRI-PDFF

Characteristics	Overweight/obesity		*P*	Lean/normal weight NAFLD		*P*	Type-2 diabetes		*P*
	**-SHUA** n=471(72.9%)	**+SHUA** n=175(27.1%)		**-SHUA** n=108(83.1%)	**+SHUA** n=22(16.9%)		**-SHUA** n=212(71.6%)	**+SHUA** n=84(28.4%)	
Age, years	41.0±11.1	37.7±13.3	<0.01	43.7±12.9	39.0±10.4	0.12	51.6±12.3	44.8±14.0	<0.01
Male, n(%)	370(78.6)	139(79.4)	0.87	64(59.3)	15(68.2)	0.16	167(78.8)	49(58.3)	<0.01
BMI, kg/m^2	27.2±3.0	28.3±3.6	<0.01	21.6±1.2	21.7±1.2	0.71	26.5±2.8	27.4±2.9	<0.01
Waist circumference, cm	90.9±7.8	93.7±8.9	<0.01	78.1±5.4	79.5±3.8	0.28	91.3±8.0	93.0±8.5	<0.01
Abodominal obesity, n(%)	283(60.1)	133(76.0)	<0.01	11(10.2)	2(9.1)	0.68	138(65.1)	69(82.1)	<0.01
WHR	0.9±0.05	0.9±0.05	0.04	0.8±0.05	0.9±0.04	0.46	0.9±0.06	0.9±0.05	0.12

SBP, mmHg	130±15	134±15	<0.01	126±17	126±19	0.96	131±16	135±19	<0.01
DBP, mmHg	83±11	86±11	<0.01	80±12	83±12	0.28	82±12	84±13	0.11
Hypertension,n(%)	127(27.0)	80(45.7)	<0.01	34(31.5)	6(27.3)	0.70	71(36.3)	44(52.4)	<0.01
ALT, U/L	38±27	42±29	<0.01	35±27	32±21	0.57	35±26	39±32	<0.01
AST, U/L	26±22	34±38	<0.01	37±33	35±13	0.69	29±25	37±31	<0.01
GGT, U/L	59±66	66±45	<0.01	57±77	59±73	0.91	72±58	78±61	<0.01
ALP, U/L	79±24	86±72	0.04	77±21	81±17	0.38	76±17	96±70	<0.01
Total cholesterol, mmol/L	5.1±1.0	5.3±1.0	0.02	5.0±1.2	4.9±0.9	0.53	5.0±1.2	5.2±1.4	<0.01
Triglyceride, mmol/L	1.7±0.9	2.2±1.5	<0.01	1.6±0.9	1.7±1.0	0.71	2.0±1.0	2.6±1.4	<0.01
HDL-C, mmol/L	1.1±0.3	1.1±0.2	0.07	1.3±0.4	1.2±0.3	0.32	1.2±0.3	1.1±0.3	0.25
LDL-C, mmol/L	3.3±0.8	3.4±0.7	0.09	3.1±0.8	3.0±0.8	0.69	3.2±0.9	3.1±0.9	0.36
FFA, µmol/L	538±177	596±266	<0.01	561±191	584±143	0.61	563±208	632±184	<0.01
FBG, mmol/L	4.9±0.6	4.9±0.7	0.94	4.8±0.6	4.7±0.9	0.31	7.8±1.6	8.1±3.2	0.70
HbA1c, %	6.1±1.7	6.2±2.0	0.18	5.9±1.6	6.0±2.3	0.71	7.2±1.8	7.3±2.7	0.31
FINS, µU/ml	11.8±10.3	13.6±8.4	0.02	8.8±4.6	8.6±3.4	0.77	15.1±9.4	13.6±5.4	0.54
HOMA-IR	2.7±2.4	3.0±2.0	0.03	1.9±1.0	1.8±0.8	0.51	5.5±4.0	5.2±3.0	0.52

HOMA- IR≥2.5, n (%)	152(32.3)	71(40.6)	<0.01	28(25.9)	3(13.6)	0.47	132(62.3)	70(83.3)	<0.01
Uric acid, μmol/L	384±67	544±74	<0.01	348±61	523±56	<0.01	366±65	516±78	<0.01
FIB-4	0.8±0.5	0.9±0.6	0.07	1.2±1.0	1.0±0.5	0.33	1.1±0.7	1.0±0.6	0.41
FIB-4 > 1.30, n(%)	67(14.2)	21(12.0)	0.54	25(23.1)	5(22.7)	0.73	65(30.7)	24(29.3)	0.74
SWE, kpa	6.0±3.0	6.1±3.1	0.87	6.1±3.4	5.7±2.9	0.67	6.2±3.2	6.0±2.3	0.76
SWE≥6.1 kpa, n(%)	225(47.8)	86(49.1)	0.76	46(42.6)	9(40.9)	0.88	112(52.8)	45(53.6)	0.76
Liver fat content, %	13.0±7.1	17.3±8.5	<0.01	11.1±6.5	15.6±7.9	<0.01	14.5±6.9	20.1±9.6	<0.01
Moderate to severe steatosis, n(%)	102(21.7)	85(48.6)	<0.01	20(18.5)	8(36.4)	<0.01	72(34.0)	43(51.2)	0.28
Severe steatosis, n(%)	50(10.6)	43(24.6)	<0.01	9(6.5)	5(13.6)	0.38	39(18.4)	35(41.7)	0.02

Abbreviation: NAFLD, nonalcoholic fatty liver disease; MAFLD, metabolic-associated fatty liver disease; SHUA, super hyperuricemia; BMI, body mass index; WHR, waist-to-hip ratio; SBP, systolic blood pressure; DBP, diastolic blood pressure; T2DM, type 2 diabetes mellitus; ALT, alanine aminotransferase; AST, aspartate aminotransferase; GGT, γ-glutamyl transpeptidase; ALP, alkaline phosphatase; HDL-C, high-density lipoprotein cholesterol; LDL-C, low-density lipoprotein cholesterol; FFA, free fatty acids; FBG, fasting blood glucose; HbA1c, glycated hemoglobin;HOMA-IR, homeostasis model assessment of insulin resistance; FIB-4, fibrosis-4 index; APRI: the AST to Platelet Ratio Index;SWE, shear wave elastography.
